# UnderstAID, an ICT Platform to Help Informal Caregivers of People with Dementia: A Pilot Randomized Controlled Study

**DOI:** 10.1155/2016/5726465

**Published:** 2016-12-28

**Authors:** Laura Núñez-Naveira, Begoña Alonso-Búa, Carmen de Labra, Rikke Gregersen, Kirsten Maibom, Ewa Mojs, Agnieszka Krawczyk-Wasielewska, José Carlos Millán-Calenti

**Affiliations:** ^1^Research, Development and Innovation Department, Gerontological Complex La Milagrosa, Provincial Association of Pensioners and Retired People (UDP) from A Coruña, Avenida de Cadiz-5, 15008 A Coruña, Spain; ^2^Center for Research in Ageing and Dementia, Faculty of Health Sciences, VIA University College, 8200 Aarhus, Denmark; ^3^Department of Clinical Psychology, Poznan University of Medical Sciences, 70 Bukowska Street, 60-812 Poznan, Poland; ^4^Department Rheumatology and Rehabilitation, Poznan University of Medical Sciences, 135/147 28 Czerwca 1956r. Street, 61-545 Poznan, Poland; ^5^Ortopedyczno-Rehabilitacyjny Szpital Kliniczny nr 4 im. W. Degi., Poznań, Poland; ^6^Gerontology Research Group, Department of Medicine, Faculty of Health Sciences, Universidade da Coruña, Campus de Oza, 15071 A Coruña, Spain

## Abstract

Information and communications technology (ICT) could support ambient assisted living (AAL) based interventions to provide support to informal caregivers of people with dementia, especially when they need to cope with their feelings of overburden or isolation. An e-learning platform (understAID application) was tested by informal caregivers from Denmark, Poland, and Spain to explore the technical and the pedagogical specifications, as well as evaluating the impact of its use on the psychological status of the participants. 61 informal caregivers completed the study taking part in the experimental (*n* = 30) or control (*n* = 31) groups. 33.3% of the caregivers were satisfied with the application and around 50% of the participants assessed it as technically and pedagogically acceptable. After using understAID the caregivers in the experimental group significantly decreased their depressive symptomatology according to the Center for Epidemiologic Studies Depression scale, but a possible benefit on their feelings of competence and satisfaction with the caring experience was also observed. The low scores obtained for satisfaction were highlighting issues that need to be modified to meet the informal caregivers' needs in national, social, and cultural context. Some possible biases are also considered and discussed to be taken into account in future improvements of understAID application.

## 1. Introduction

Global prevalence of dementia is growing as life expectancy increases and demographic ageing occurs. Almost 47 million people worldwide were living with dementia in 2015, 10.5 million corresponding to the European Union [1]. This increased prevalence implies an important impact on families, health care services, and overall costs of the society. People living in the community with dementia are in most of the cases cared by informal caregivers, mainly spouses or adult children, mostly female [2] but there are wide differences among countries across Europe. These differences reflect not only cultural preferences and sociodemographic factors but also public policies in the long-term care systems [3, 4].

Informal caregiving is an important factor not only for preventing the institutionalization of the elderly but also for avoiding adverse health outcomes [5]. In this sense, informal caregivers show a great incidence of health problems both psychological such as depression, high levels of neuroticism, anxiety symptoms, sleeplessness and cognitive problems [6–9] and medical such as cardiovascular diseases [10]. In addition, informal caregiving tends to be seen as cost-effective from a public economic point of view. However, this perspective does not take into account the indirect costs produced by the negative impact on employment and health in the caregivers [3].

Provision of support is essential to sustain the caregiving responsibilities, to prevent the burden, and to reduce the depressive symptomatology of informal caregivers [11]. Thus, it is important that social and sanitary services develop cost-effective strategies that improve the quality of life for both caregivers and patients with dementia. In this sense, psychoeducational interventions providing skill training and education to carers have shown improvements in emotional state and level of stress of caregivers [12–14]. In fact, it has been suggested that coping strategy based support is especially helpful for informal caregivers given that a “dysfunctional coping” predicts depression and anxiety in carers [14]. Particularly, the development of information and communications technology (ICT) has become a promising tool for family caregivers of people with dementia, by improving the caregivers' subjective wellbeing [15–18] but also by teaching them better ways of coping with the stress of caregiving [17]. ICT can support ambient assisted living (AAL) interventions to enable older adults to age at their own home and their communities. In fact it has been already suggested that AAL interventions not only could assist the informal caregivers and their relatives with dementia, but also can alleviate their transition to a situation of greater dependency while the disease evolves [19].

These kinds of interventions can assist informal caregivers by offering them relevant education, support, and training at home with a relative low cost and with the possibility of customizing the activities and contents they wish to consult or even when they would like to obtain this support according to their availability, preferences, priorities, or emotional state [19].

Several studies show that ICT interventions are evaluated by carers as useful, educational, and convenient [20–23]. Furthermore, in the recent review carried out by McKechnie et al. [24], the authors found that computer-mediated interventions are generally positive and can reduce effectively carer burden, anxiety, and depression. Nevertheless, these effects were moderated by characteristics of participants such as the level of mastery or depression.

Following the web-based education programs and AAL interventions, the understAID application was developed with the aim of helping informal caregivers of people suffering from dementia, by providing them with information, skills training, a forum to develop their social network, and daily reminders. Based on the review of McKechnie et al. [24], we hypothesized that the use of this application would reduce the depressive symptomatology and improve the wellbeing and coping skills of the relatives and according to previous research [19] it would have a positive impact on their competence and confidence as caregivers by complementing the care provided by them. This study had different aims: (a) to test the technical and pedagogical specifications of understAID, (b) to assess the satisfaction of the caregivers with the application, and (c) finally to evaluate the impact on the psychological status of the participants.

## 2. Method

### 2.1. Study Design

A pilot randomized controlled pre-post intervention trial was conducted to establish the effectiveness of the understAID application among informal caregivers of people with dementia. Participants were randomly assigned to the experimental or to the control group by using a computer-based random number generator.

### 2.2. Participants and Recruitment

Participants were 103 informal caregivers taking care for someone diagnosed with dementia according to the Global Deterioration Scale (GDS) [25] and were living in either Denmark, Poland, or Spain. Many of them formerly participated in the previous selection of topics to be included in the understAID application (see Section 2.3 for a deeper explanation). They were recruited again from different local Alzheimer's associations of adult day-care centers: from the Danish Alzheimer Association (DAA) and the Skanderborg municipality (SKAN) in Denmark, from Poznan, Walcz, Ciechocinek, and Koszalin in Poland, and from the Gerontological Complex La Milagrosa, Saraiva-Marín, and Afal-Ferrolterra in Spain. The inclusion criteria for the study were as follows: (1) taking care of a person diagnosed with dementia by a specialist or a neurologist, according to the criteria of the Classification of Mental and Behavioural Disorders, 10th revision [26], or the Diagnostic and Statistical Manual of Mental Disorders, 4th edition, text revision [27], or the National Institute of Neurological Disorders and Stroke-Alzheimer Disease and Related Disorders Association [28]; (2) being the primary caregiver in the following aspects: executing basic care tasks for a minimum of 6 weeks, receiving no remuneration for caregiving service (except from a few Danish caregivers receiving economical compensation for reducing their ordinary working hours while caring for their demented relative), and devoting much time to patient care [29]; (3) suffering a burden according to the 22-item version of the Zarit Burden Interview [30], using a cut-off point of 24, which was determined to identify family caregivers who are at risk for depression [31]; and (4) signing the informed consent form to participate in the study. The exclusion criteria were to present some of the following conditions that might prevent the evaluation of the participant or the interaction with the platform: cognitive impairment, illiterate, severe hearing and visual or motoric problems.

### 2.3. The UnderstAID Application

The understAID application was developed to be accessible through any device with Internet connection: by using an application in any mobile device (Smartphone or Tablet) or through a browser in a personal computer (PC). The understAID consists on a Learning section with a database of contents organized in 5 modules with information about 15 different topics. The topics cover information about the care of a person with dementia and caring for oneself as a caregiver. The topics consist of text, videos, and images and they also include references to other websites. The modules and topics included in understAID are Module 1, Cognitive Declines (Topics: Attention, Memory, and Orientation); Module 2, Daily Tasks (Topics: Bathing, Incontinence, Massage and Touch, and Physical Exercises); Module 3, Behavioural Changes (Topics: Anxiety and Agitated Behaviour, Depressive Mood, Manic Symptoms, and Emotional Control and Recognition); Module 4, Social Activities (Communication and Apathy and Loss of Motivation); and Module 5, You as a Caregiver (Topics: Coping with Own Stress and Motivation). In addition, the information of each topic is organized in four levels of complexity starting by quick fixes level, followed by simple insight, explanations, and finally more details level.

It also contains a Daily Task section with the option of using a calendar and reminders for appointments and medication intake. Additionally, it has a Social Network section where the caregivers can interact with other participants and exchange information and opinions. This section was moderated by the researchers of the study.

The understAID also includes the option of filling in an interactive customization questionnaire with questions about the level of dementia of the person cared by the informal caregiver and about the preferences, energy, and time availability for learning of the care provider. By answering these questions at the entry of the application, the information level showed to the informal caregivers is personalized and adjusted to their personal situation.

For the final selection of topics to be included in the Learning section, 101 informal caregivers of patients with dementia living in the community were previously recruited from local Alzheimer's associations or adult day-care programs from 3 countries: Spain, Poland, and Denmark. The caregivers were evaluated using a comprehensive assessment by clinical professionals though a self-elaborated questionnaire collecting sociodemographic variables. As a result, the age range of caregivers was 25–88 years; the majority of caregivers were female with a medium level of education (46.5%) and married or living with someone (82.2%). Only 19.8% of the caregivers were taking psychoactive drugs. Regarding the type of relationship between the primary caregiver and the person with dementia, there was a similar proportion of caregivers with no consanguinity (47.8%) and with consanguinity (52.2%) relationship with the care recipient. There was also the same amount of employed and unemployed caregivers (50% in each group). Additionally, the mean duration of caregiving was 64.26 months. A substantial number of care recipients suffered from severe or very severe cognitive impairment (GDS6 and GDS7, 56.4%) with a 53% diagnosis of patients diagnosed with Alzheimer's disease. For the selection of topics, the caregivers were asked to fill in a questionnaire about daily situations they might encounter while taking care of their relatives with dementia. The frequency and severity of the patient behaviour along with the discomfort that this behaviour caused in the caregiver were collected and analysed to finally select the 15 topics included in the understAID application.

### 2.4. Procedure

Once the caregivers expressed their desire to participate, they were provided with extra written and oral information along with a consent form. When the signed informed consent form was obtained, the participants received the preintervention questionnaires (to collect the characteristics of the study sample and the understAID psychological impact on the caregivers) to be self-completed and returned to the researchers.

Participants in the experimental group were provided with a link to download the understAID application in their mobile devices (Smartphone or Tablet) and also a link to use the application through the browser in their PCs. They were instructed to use the application, browse the different topics, visit the different sections, and watch the diverse media contents. Participants in the control group did not use the application and maintained their usual lifestyle. According to previous studies based on ICT training programs, the period for the pilot testing was established in three months [17]. During this period, the research staff from each country did a follow-up of the caregivers' performance through phone calls on a weekly or monthly basis. Verbal information regarding the troubles or opinion about the application was collected from the caregivers. At the end of the three-month period, all participants were given the postintervention questionnaires to be self-completed and returned to the researchers (technical, pedagogical, and satisfaction questionnaires along with the questionnaire to collect the psychological impact of understAID on the caregivers). All personal data collected were anonymized by assigning a code number to each participant. This codification information was saved in a database under supervision of the project leaders in each location.

The study protocol was approved by the local ethics committees in all three countries according to the specific national guidelines and conformed to the principles of the Declaration of Helsinki. Before data collection, all of the participants were provided with information concerning the study and signed the informed consent.

### 2.5. Variables and Instruments

All questionnaires were provided to the caregivers to be filled either online or hand written. They were self-completed by the participants in their own language.

#### 2.5.1. Characteristic of the Sample

The characteristics of the study sample were gathered through a self-elaborated preintervention questionnaire that included information about gender, country of origin, occupation (physical labour, intellectual labour, unemployed, and retired), loss of professional career, change in working shift, frequency of caring (<20 hours per week; ≥20 hours per week), kind of support received, feelings of institutionalization of the person with dementia (never, sometimes, and frequently), self-perceived health (very good, good, fair, and poor), and visits to the general practitioner in the last month. The level of burden due to the caregiving tasks was assessed through the Zarit Burden Interview (ZBI) [30]. The maximum score in this scale is 88 points, with higher scores indicating an increased level of caregiver burden.

The Global Deterioration Scale (GDS) [25] was used to establish the severity of dementia of the person in charge. This scale classifies the cognitive function into seven stages, from no cognitive decline to very severe cognitive decline, also including aspects related to functional and behavioural domains. The levels 4 and 5 indicate mild and moderate dementia, whereas levels 6 and 7 indicate moderately severe and severe dementia. Only informal caregivers of patients classified as GDS 4, 5, 6, or 7 were included in the study.

#### 2.5.2. Assessment of the UnderstAID Feasibility

Feasibility was evaluated during the postintervention assessment of the experimental group with three self-completion questionnaires developed for this study. All the questions included in these questionnaires are shown in Tables 2, 3, and 4 in the results section. These questionnaires aimed at assessing the technical specifications of the application (technical questionnaire), the pedagogical aspects of the application and its contents (pedagogical questionnaire), and the general satisfaction of the caregiver after using the application (satisfaction questionnaire). The technical questionnaire contains 11 questions to assess the caregivers' opinions about technical aspects of the application. The pedagogical questionnaire contains nine sections with a total of 20 questions to assess the caregivers' opinions about pedagogical aspects of the application. Finally, the satisfaction questionnaire contains 5 questions to assess the caregivers' satisfaction about the application. A 5-point Likert scale ranging from 1 (strongly disagree) to 5 (strongly agree) was used to score each of the questions in every questionnaire. For the individual items of the questionnaires, mean scores and standard deviation were calculated. Mean items scores of 4 (agree) and higher were considered positive aspects. Mean items scores of 2 (disagree) and 1 (strongly disagree) were considered as negative aspects and will need further revisions.

#### 2.5.3. Assessment of the Psychological Impact of UnderstAID on Caregivers

For assessing the psychological impact that the use of the understAID application may have on the caregivers, three scales were applied before and after the intervention to all the participants (control and experimental groups). Depressive symptoms were measured using the Center for Epidemiologic Studies Depression Scale (CES-D) [32]. All items are rated on a 4-point Likert-type frequency scale, ranging from 0 (rarely or none of the time) to 3 (most or all of the time). The total score of the 20 items ranges from 0 to 60, with higher scores indicating more depressive symptoms severity. The sense of competence experienced by the caregiver was measured using the Caregiver Competence Scale (CCS) [33]. This scale, composed of 4 items, measures the caregivers' perceived competence of their own performance in the caregiving tasks, with a maximum score of 16 points and higher scores indicating more feelings of competence: (1) acquired knowledge to deal with difficult situations; (2) overall feelings of being a good caregiver; (3) feelings of competence; and (4) feelings of self-confidence. Finally, the caregiving satisfaction with the care provided was assessed using the Revised Caregiving Satisfaction Scale (RCSS) [34]. This scale is composed of 6 items, evaluating different positive aspects of caregiving: (1) global satisfaction helping the relative; (2) feeling closer to the patient; (3) enjoying being with the patient; (4) boosting the caregiver's self-esteem; (5) delighting in the patient's pleasure; and (6) giving meaning to the caregiver's life. With a maximum score of 30 points, higher scores in the RCSS indicate more feelings of satisfaction.

### 2.6. Statistical Analysis

The quantitative variables were shown as mean ± standard deviation (SD). The qualitative variables were expressed as a percentage. The normality of the quantitative data was tested using the Kolmogorov-Smirnov test. Differences in the distribution of the baseline characteristics between control and experimental groups were examined using chi-square tests for qualitative variables and Student's *t*-test for quantitative variables. Differences in the outcomes of the scales before and after the intervention were examined using the paired sample test. A significance value of 5% was accepted for all the analysis. Statistical analysis was performed using the software package SPSS v. 15.0 (SPSS Inc., Chicago, IL, USA).

## 3. Results

### 3.1. Recruitment and Description of Participants

A total of 103 participants were recruited among the three participating countries. After applying the inclusion and exclusion criteria, the initial sample was reduced to 77 caregivers that completed the initial assessment and started the pilot testing period. Participants were distributed randomly in control (*n* = 41) and experimental (*n* = 36) groups.

**Figure 1:**
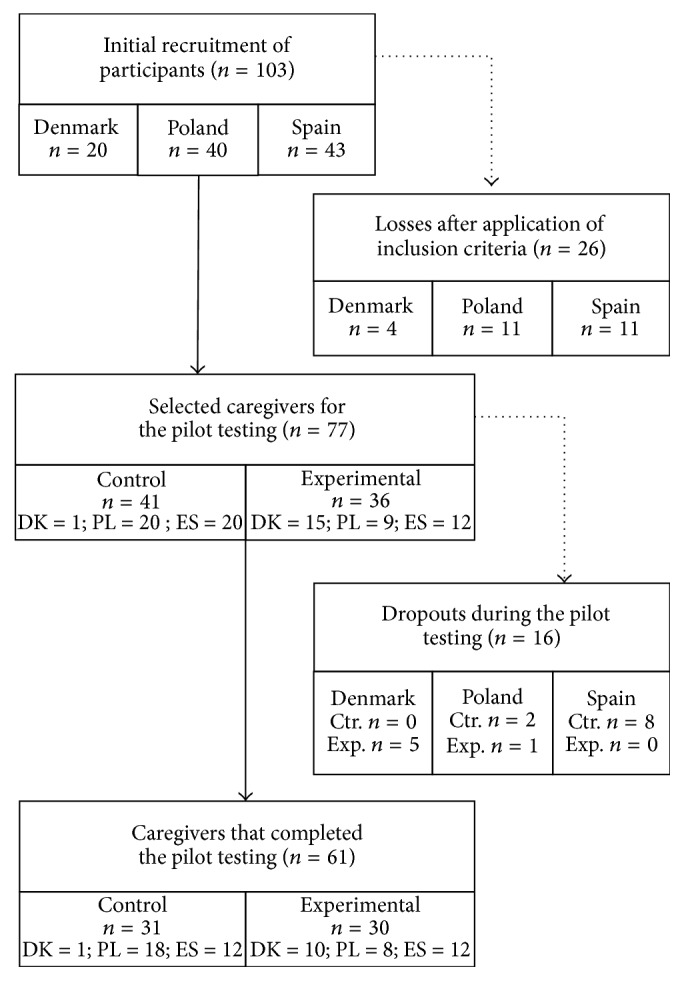
Flow of selection for the participants. Ctr.: Control, DK: Denmark, ES: Spain, Exp.: Experimental, PL: Poland.

During the three months of intervention, 16 caregivers dropped out due to different reasons. These reasons were finding uselessness of the application (Denmark, experimental group, *n* = 1), suffering a disease (Denmark, experimental group, *n* = 1), lack of time for using the application (Denmark, experimental group, *n* = 1), or not completing the final assessment despite the repeated request by researchers to fill in the questionnaires (Denmark, experimental group, *n* = 2; Poland, experimental group, *n* = 1; Poland, control group, *n* = 2; Spain, control group, *n* = 8). Eventually, the total number of caregivers that completed the pilot period was 61 and was distributed as follows: 31 caregivers in control group and 30 in experimental group (Figure 1).

Regarding the basal characteristics of the study sample (Table 1), they were mainly women (63.9%), performing an intellectual labour (41.0%) and although only 14.8% of the caregivers gave up their professional career, 37.7% were forced to change their working hours. No differences were observed regarding the number of hours dedicated to caregiving tasks and almost 100% of the participants were receiving some kind of support for these duties. The most frequent forms of support used were relative and friends (44.3%), day-care center (44.3%), general practitioner (39.3%), and respite units (31.1%). There were statistically significant differences regarding the dementia supervisor (*P* = 0.020) and the respite care (*P* = 0.016).

Regarding the self-perceived health, most of the caregivers assessed their health as good (41.0%) or fair (47.5%) with a very low percentage of the option poor selected (1.6%). Visits to the general practitioner during the last month before the assessment were also infrequent, with more than half of the caregivers (57.4%) paying no visit to their doctors.

With regard to the severity of dementia of the cared-for patients, the most frequent GDS stages were GDS4 (31.1%) and GDS5 (36.1%), followed by GDS6 (26.2%) whereas only 6.6% were severely demented (GDS7).

At baseline, in both groups there were no statistically significant differences according to ZBI results (47.55 ± 10.02 in control versus 42.20 ± 11.46 in experimental group). Furthermore, no differences were observed in those psychological characteristics that might be affected after the use of the understAID application such as depressive symptomatology (21.42 ± 8.64 in control versus 19.40 ± 9.03 in experimental group), sense of competence (11.03 ± 2.24 in control versus 11.93 ± 2.61 in experimental group), and caregiving satisfaction (19.55 ± 6.40 in control versus 18.67 ± 4.90 in experimental group).

### 3.2. Evaluation of UnderstAID Feasibility

The assessment of the technical specifications according to the device used is shown in Table 2. The participants scored higher the items of the technical questionnaire when the device used was the PC. This tendency was observed for all the sentences except for “the application responds quickly and smoothly” and “the help options are useful.” The best scores were obtained for the sentences “the position of the images is clear in the application” (4.07 ± 1.02) and “the contrast between colours (texts on a background, colours used in images) and the text size makes the application easy to use” (4.17 ± 0.99). In the overall evaluation scores of technical specifications, for PC and Tablet the percentage of caregivers that assessed the specifications as acceptable was almost equal to the percentage of caregivers assessing them as unacceptable. For the case of Smartphone device use, most of the caregivers (71.4%) assessed the specifications as unacceptable. The percentage of caregivers assessing the application as technically acceptable (46.7%) was almost equal to the ones assessing it as unacceptable (53.3%).

With regard to pedagogical aspects of the understAID application (Table 3), the lower scores (less than 3) were given to the issue “I have used the search function” from Section 1 (Way of Obtaining the Information), and to Sections 5 (Content), 6 (Confidence), 7 (Motivation), 8 (Social Learning), and 9 (Daily Tasks). Nevertheless, the highest score in Section 1 was given to the sentence about the use of the customization questionnaire “I have filled in the customization questionnaire and let the application suggest content” (4.23 ± 0.99) and in Section 5 to the sentence “the application has helped me understand better the illness as well as the current situation and needs of my relative” (3.44 ± 1.25). With regard to the overall assessment of pedagogical specifications, there was a draw between acceptable (50%) and unacceptable (50%) scores.

Finally, with regard to the general satisfaction of the caregivers with the application (Table 4), the scores given to every item of the satisfaction questionnaire were very low and this result was coherent with the observed overall evaluation of the satisfaction, where 66.7% of the caregivers assessed the understAID application as unacceptable.

### 3.3. Evaluation of the Psychological Impact of UnderstAID on Caregivers

Comparison of CES-D, CCS, and RCCS scores before and after the three-month intervention showed no differences in the control group (Table 5). In the experimental group, there were statistically significant differences for the CES-D score pre- versus postintervention (19.40 ± 9.03 versus 17.03 ± 7.07, *P* = 0.037), with a decrease in the observed depressive symptomatology.

With regard to the individual items for CCS, the highest scores given by the caregivers of the experimental group after the intervention were assigned to point 1 (acquired knowledge to deal with difficult situations; 3.10 ± 0.76) and point 2 (overall feelings of being a good caregiver; 3.07 ± 0.70) followed by point 3 (feelings of competence; 2.80 ± 0.81) and finally point 4 (feelings of self-confidence; 2.73 ± 0.79).

Lastly, with regard to the specific items for RCSS, the highest scores were given to point 1 (global satisfaction helping the relative; 3.50 ± 1.14), point 5 (delighting in the patient's pleasure; 3.50 ± 0.73) and point 3 (enjoying being with the patient; 3.43 ± 0.94) followed then by point 2 (3.00 ± 1.17), point 6 (2.60 ± 1.13), and finally point 4 (2.57 ± 1.07).

## 4. Discussion

The understAID platform, an online e-learning application created to help informal caregivers of people with dementia through ambient assisted living interventions (AAL), was evaluated in Denmark, Poland, and Spain on its technical and pedagogical specifications along with the satisfaction and impact on the caregivers who used it. The evaluation was done in a randomized controlled study of three months, comparing the participants who trained with the understAID application to a control group that did not interact with the learning platform.

Most of the caregivers that participated in the study were female, in coherence with the results of previous works [35] and with the fact that the care of the relatives used to rely on the female individuals of the family [2, 36]. The high percentage of actively working caregivers (62.3%) points to the fact that many of them are juggling their working and caring duties. This interpretation is supported by the 37.7% of change in their working hours. Additionally, almost all the caregivers counted on some kind of support for their caring activities. Among the different options, the most frequent was receiving support from other family members or friends and from a day-care center. The differences observed regarding the support from the dementia supervisor were due to the fact that this is a professional key support concept in municipalities only existing in Denmark, where almost all the caregivers were randomly allocated in the experimental group. Something similar happened with the support from the respite units, mainly used by Polish caregivers that were also randomly allocated in the control group with a high percentage. Despite this sort of support, more than 50% of the caregivers expressed their feelings about institutionalizing their relatives with dementia which is a measure of the burden they are feeling due to the caring activities. In fact, all the caregivers participating in the study were suffering burden according to ZBI. Anyhow, expressing this desire is only a declaration of intentions that many times cannot be executed due to the economic crisis that affects worldwide, hence the need of creating new tools to ease the duties of these caregivers in a cost-effective way and consequently improving their quality of life. Information and communications technology (ICT) in the frame of AAL interventions is a promising treatment not only for people suffering from cognitive decline, thus slowing the evolution to dementia [37], but also for patients with dementia and their informal caregivers [19]. According to a recent review of the emerging role of smart technologies in dementia care, patients can benefit by maintaining their cognitive skills and social interaction while the informal caregivers are the vast majority target group for telemedicine [38] as it happens with the understAID application whose main target group are the informal caregivers of people with dementia. In addition, ICT have been suggested as a suitable tool to teach the caregivers better ways of coping with the stress of caregiving [17].

Caregivers that tested the application found some technical troubles that were reported to the research team to be corrected in the future version of understAID and it may be well possible that these initial difficulties triggered the noncompleters rate of around 20%. In line with previous works, we receive critics related to the user-friendliness of the application (navigation through the menu, help and search options, not being very intuitive, and so on) [39], but when observing the results by device, we found out that the lowest scores were given by the Smartphone users. This outcome might be explained by two facts. Firstly, the use of ICT is not completely extended in the population, finding important differences regarding the nation of origin. According to recent statistics, Denmark is the country where the Internet usage is higher (84% of people using Internet everyday) compared to Spain (54%) and Poland (47%) [40], which might be well an image of the caregivers' skills to use mobile devices and accordingly about their negative opinion on the technical characteristics. In the study population, the percentage of caregivers in the experimental group from Poland and Spain (66.7%) doubled the percentage of Danish participants (33.3%), which in fact might be biasing the outcomes. And secondly, the screen size in the Smartphone devices (smaller than in Tablets of PC) was also one of the main complaints received by the participants that could not see properly the media files associated with the topics, thus possibly affecting the caregivers' opinion.

Regarding the answers given by the caregivers to the pedagogical questionnaire, the participants found it very useful to personalize the information by using the customization questionnaire at the beginning of the logging procedure in the application. This result is in concordance with previous publications about the possibility of customizing the activities [19] or even the information by using an interactive learning advisor [41]. This personalization is very convenient given that it allows the caregivers to tailor not only the content but also the level of information according to their individual needs and time allowance. Although very few caregivers used the search option in the application, it may be well explained by the fact that, due to being a prototype testing, the amount of contents was not very high. Nevertheless, it is expected that, with higher numbers of contents, this option would be more useful. Most of the caregivers found not completely useful the information received to help taking care of their relatives in a better way or making them feeling more confident with the care given and increasing their coping strategies. As previous works reported, their opinion about the application content was that it would be better attuned to the needs of a caregiver with a relative in the early stages of dementia [23]. This might be consequence of their broad knowledge with their caring duties, given that all of them were experienced caregivers with years of learning baggage about how to cope with the care of their relative. This result is in concordance with previous works that did not find differences among pretest-posttest intervention with a multimedia support program, where the caregivers indicate no significant improvements in their frequency of employing specific coping skills [18]. Nevertheless, this lack of effect might be due to the short length of intervention in this study (three months), not enough to find a significant effect of the understAID use. However, in line with previous studies, they did find the application useful to better understand the disease and the current situation and specific needs of their relatives [23]. This result is very interesting given that it is pointing out to the fact that many caregivers still did not comprehend the basics of dementia and might be fighting against the behaviour of their relatives instead of assuming the way the disease develops. Knowing the peculiarities of dementia disease would help the caregivers to increase their empathy for the relative, viewing the disease from the point of view of the patient, thus helping them to provide a better care [41]. These outcomes suggest that ICT approaches to educate and teach the informal caregivers might be the solution to help them manage their stress due to the day-to-day demands of their diseased relatives. Additionally, it would allow them to be prepared for the following stages of dementia when the level of dependency will be higher [19].

As happened in other studies about web-based programs for caregivers, the social network section was negatively reviewed as a forum place [42]. According to the feedback received verbally by the caregivers, this section was scarcely used but to our understanding, the low scores given to the social network section are the reflection of the needs to share their knowledge, tips, feelings, desires, and so forth. As in many other situations, the caregivers expressed their desire of obtaining or sharing information with other informal peer caregivers in the very same situation as themselves [23], instead of only reading contents developed by a panel of experts. Based on this, the social network section improvement must be a priority in the implementations to be done to the understAID application in the future. With regard to the lack of free time after using the understAID application, this result is in concordance with previous studies that discovered that caregivers found the supporting applications too time consuming given that they cannot be integrated with their existing medical records [39]. In line with this result are the low scores given to Section 9 (Daily Task) of the application since the caregivers would have wished to synchronize the calendar with the one from their own device.

Altogether, the technical bugs that happened during the pilot testing plus the pedagogical deficiencies might be the origin for the low satisfaction with the application expressed by the caregivers. But, the most important part of the negative feedback received is that it is pointing out to the characteristics of the application that needs to be implemented and improved so the quality of the information for the caregivers is the better and best delivered.

The use of the application positively impacted the caregivers by reducing their depressive symptomatology. This result is in line with previous works that found that depressive symptoms were decreased by the use of technology based interventions [16]. A possible explanation might be that, after using the application, many of the caregivers realized that their doubts, feelings, or frustrations are common among informal caregivers of patients with dementia, thus encouraging them to constructively assume their situation and reduce their emotional distress. Additionally the information provided by the app might be increasing the self-efficacy in the caregivers and finally decreasing their depressive symptoms. Beneficial effects of information provision have been previously obtained by other authors [43] as well as increases in the self-efficacy by using Internet multimedia programs [18].

No change was observed in their feelings about the competence and satisfaction as caregivers, but this is coherent with the fact previously commented about their years of experience with the care of the relative with dementia. It is expected that they are mastered in the caring duties and coping strategies so any improvement due to the use of the application would be insignificant. Consequently, the application could be tested in the future by unexperienced caregivers at the very beginning of their caring experience and due to that it is expected an increase in their feelings of competence and satisfaction, given that they will benefit more from the contents about how to take care of their relatives, how to take care of themselves, how to better understand the disease, and finally how to cope with the day-to-day situations [23].

This study suffered from some kind of limitations, starting by a possible sample bias based on the caregivers access to the Internet [40], their previous experience with smart devices, or even the different specifications (such as the screen size) of the appliances used. The collection of some of the data was made by using paper questionnaires that were delivered after the end of the pilot testing, thus increasing the odds of noncompleters, the appearance of missing data, or the data entry errors. Besides, given that the questionnaires were filled in at the end of the three-month period, it may be possible that the caregivers were not accurately recalling their experience with the understAID application, so a daily or weekly monitoring would have been more informative. Additionally, a follow-up after the intervention should have been considered to assess the duration of the impact of understAID use on the caregivers over the time. The study population had also marked national differences in the support received, maybe due to the fact that there are broad differences among European countries in their health policies, being Denmark among the nations characterized by a lower provision of informal care and a high use of formal long-term care services [3]. Additionally, the follow-up contact might also be influencing the results due to two facts: the communication was only done with the experimental group, which may have had a positive effect on their decrease in the depressive symptomatology, and the frequency of phone calls was different depending on the country, being the Spanish caregivers the ones receiving more frequent calls. And finally, the control group did not receive any other alternative intervention, so they did not have to spend time on learning which may have been an additional burden for the caregivers in the experimental group and consequently affect their overall satisfaction with the application.

## 5. Conclusions

In conclusion, half of the participants who completed the intervention evaluated positively the application regarding the technical and pedagogical specifications, with some particular points that obtained lower scores. Regarding satisfaction, the scores obtained were low in general. These negatively scored issues will need to be reviewed for improving the future versions of understAID. Moreover, the results of the randomized controlled study demonstrated a significant positive impact of understAID on decreasing the depressive symptomatology of the caregivers that used the application. But additionally, it is expected that unexperienced caregivers will benefit the most of understAID by improving their depressive symptoms but also their feelings of competence and satisfaction with the caring duties and also by increasing their knowledge about how to cope with the day-to-day situations with their relatives. The development of technological applications and ambient assisted living interventions for informal caregivers has opened a wide range of new supporting possibilities so computer applications might be a useful tool to improve not only their caring and copings skills but also their wellbeing.

## Figures and Tables

**Table 1:** Characteristics of the study sample.

	Total *n* (%)	Control group *n* (%)	Experimental group *n* (%)
	61 (100.0)	31 (50.8)	30 (49.2)
*Gender*			
Male	22 (36.1)	13 (41.9)	9 (30.0)
Female	39 (63.9)	18 (58.1)	21 (70.0)
*Occupation*			
Physical labour	13 (21.3)	7 (22.6)	6 (20.0)
Intellectual labour	25 (41.0)	15 (48.4)	10 (33.3)
Unemployed	5 (8.2)	4 (12.9)	1 (3.3)
Retired	18 (29.5)	5 (16.1)	13 (43.4)
*Professional career*			
Loss of professional career (yes)	9 (14.8)	4 (12.9)	5 (16.7)
Change in working shift (yes)	23 (37.7)	13 (41.9)	10 (33.3)
*Hours of caring/week*			
<20	35 (57.4)	18 (58.1)	17 (56.7)
≥20	26 (42.6)	13 (41.9)	13 (43.3)
*Support *			
Yes	59 (96.7)	31 (100.0)	28 (93.3)
*Kind of support (yes)*			
General practitioner	24 (39.3)	12 (38.7)	12 (40.0)
Dementia supervisor	8 (13.1)	1 (3.2)	7 (23.3)^*∗*^
Relatives/friends	27 (44.3)	11 (35.5)	16 (53.3)
Home services	7 (11.5)	3 (9.7)	4 (13.3)
Telecare	1 (1.6)	1 (3.2)	0 (0.0)
Day-care center	27 (44.3)	13 (41.9)	14 (46.7)
Respite care	19 (31.1)	14 (45.2)	5 (16.7)^*∗*^
Self-help groups	1 (1.6)	0 (0.0)	1 (3.3)
Local parish	3 (4.9)	0 (0.0)	3 (10.0)
*Feelings of institutionalization*			
Never	22 (36.1)	7 (22.6)	15 (50.0)
Sometimes	23 (37.7)	9 (29.0)	14 (46.7)
Frequently	16 (26.2)	15 (48.4)	1 (3.3)
*GDS stage of the relative with dementia*			
GDS4	19 (31.1)	7 (22.6)	12 (40.0)
GDS5	22 (36.1)	10 (32.3)	12 (40.0)
GDS6	16 (26.2)	11 (35.5)	5 (16.7)
GDS7	4 (6.6)	3 (9.7)	1 (3.3)
*Self-perceived health*			
Very good	6 (9.8)	1 (3.2)	5 (16.7)
Good	25 (41.0)	16 (51.6)	9 (30.0)
Fair	29 (47.5)	13 (41.9)	16 (53.3)
Poor	1 (1.6)	1 (3.2)	0 (0.0)
*Visits to general practitioner*			
0	35 (57.4)	16 (51.6)	19 (63.3)
1	17 (27.9)	10 (32.3)	7 (23.3)
2	6 (9.8)	3 (9.7)	3 (10.0)
3	3 (4.9)	2 (6.5)	1 (3.3)
*Zarit Burden Interview (mean ± SD)*	44.92 ± 11.00	47.55 ± 10.02	42.20 ± 11.46

GDS: Global Deterioration Scale.

^*∗*^Statistically significant differences, *P* < 0.05.

**Table 2:** Evaluation of technical specifications by sentence and overall.

Technical specifications questionnaire	*n* (%)	Mean ± SD
*The position of the buttons is clear in the application*		
PC	15 (50.0)	4.20 ± 1.01
Tablet	8 (26.7)	3.25 ± 1.28
Smartphone	7 (0.0)	3.86 ± 0.38
Total	30 (100.0)	3.87 ± 1.04
*The position of the images is clear in the application*		
PC	15 (50.0)	4.40 ± 0.74
Tablet	8 (26.7)	3.63 ± 1.51
Smartphone	7 (0.0)	3.86 ± 0.69
Total	30 (100.0)	4.07 ± 1.02
*The guidance provided for the application is useful*		
PC	15 (51.7)	4.27 ± 0.88
Tablet	8 (27.6)	3.63 ± 1.06
Smartphone	6 (20.7)	3.67 ± 0.82
Total	29 (100.0)	3.97 ± 0.94
*It is easy how to use the application*		
PC	15 (50.0)	4.20 ± 0.94
Tablet	8 (26.7)	3.38 ± 1.06
Smartphone	7 (0.0)	3.86 ± 0.90
Total	30 (100.0)	3.90 ± 1.00
*It is clear what happens when you click something*		
PC	15 (50.0)	4.13 ± 0.92
Tablet	8 (26.7)	3.25 ± 1.28
Smartphone	7 (0.0)	4.00 ± 0.58
Total	30 (100.0)	3.87 ± 1.01
*The contrast between colours (texts on a background, colours used in images) and the text size makes the application easy to use*		
PC	15 (50.0)	4.47 ± 0.64
Tablet	8 (26.7)	3.88 ± 1.55
Smartphone	7 (0.0)	3.86 ± 0.69
Total	30 (100.0)	4.17 ± 0.99
*The handling of the application is well adapted to my device*		
PC	14 (48.3)	4.29 ± 0.91
Tablet	8 (27.6)	3.38 ± 1.30
Smartphone	7 (24.1)	3.71 ± 0.76
Total	29 (100.0)	3.90 ± 1.05
*It is easy to find what I need*		
PC	13 (46.4)	3.85 ± 1.14
Tablet	8 (28.6)	3.38 ± 1.06
Smartphone	7 (25.0)	3.57 ± 0.54
Total	28 (100.0)	3.64 ± 0.99
*The quality of the media files (video, sound, images) is appropriate*		
PC	12 (50.0)	3.92 ± 1.00
Tablet	6 (25.5)	3.50 ± 1.05
Smartphone	6 (25.5)	3.33 ± 0.52
Total	24 (100.0)	3.67 ± 0.92
*The application responds quickly and smoothly*		
PC	15 (50.0)	3.87 ± 1.13
Tablet	8 (26.7)	4.00 ± 1.07
Smartphone	7 (0.0)	3.57 ± 0.54
Total	30 (100.0)	3.83 ± 0.99
*The help options are useful*		
PC	13 (48.1)	3.85 ± 0.99
Tablet	8 (29.6)	3.88 ± 0.99
Smartphone	6 (22.2)	4.00 ± 0.63
Total	27 (100.0)	3.89 ± 0.89

Overall evaluation of technical specifications	Acceptable *n* (%)	Unacceptable *n* (%)

PC	8 (53.3)	7 (46.7)
Tablet	4 (50.0)	4 (50.0)
Smartphone	2 (28.6)	5 (71.4)
Total	14 (46.7)	16 (53.3)

PC: personal computer, SD: standard deviation.

**Table 3:** Evaluation of pedagogical specifications by sentence and overall.

Pedagogical specifications questionnaire	*n* (%)	Mean ± SD
*Section* *1: how have you obtained information in the system?*		
It has been easy for me to find the materials that I need	27 (90.0)	3.48 ± 0.98
I have filled in the customization questionnaire and let the application suggest content	26 (86.7)	4.23 ± 0.99
I have used the search function	25 (83.3)	2.88 ± 1.30
I have browsed for content (looked at list of contents)	29 (96.7)	3.72 ± 1.16
*Section* *2: the materials have matched*		
My needs related to the care of my relative	30 (100.0)	3.40 ± 1.22
My own life situation	28 (93.3)	3.29 ± 1.21
My preferences for learning and working with the application (I prefer to learn with texts, or audio, etc.)	28 (93.3)	3.25 ± 1.18
My level of energy and resources to learn something new right now	29 (96.7)	3.24 ± 1.02
*Section* *3: adaptation to my needs*		
I have used the application's possibilities to consult contents adapted to my relative or to my desires for reading/learning new contents as well as customizing my learning experience (different detail levels, dementia stages, personal notes in contents, and so on)	30 (100.0)	3.17 ± 1.21
*Section* *4: keeping track*		
I have found it useful that my “last visited content/page” is presented to me on each entry to the application	27 (90.0)	3.63 ± 1.18
I have found it useful that my previous actions in the application are shown on entry	25 (83.3)	3.48 ± 1.30
*Section* *5: Content*		
The application helped me to overcome challenges I experience in my daily life when dealing with my relative	26 (86.7)	2.92 ± 1.09
The application helped me to overcome challenges I experience in my daily life in relation to my own life situation	26 (86.7)	2.77 ± 1.11
The application has helped me understand better the illness as well as the current situation and needs of my relative	27 (90.0)	3.44 ± 1.25
The application has provided me with all the information that I needed	27 (90.0)	2.85 ± 1.13
*Section* *6: Confidence*		
After using the application, I have become more confident about the care and help I give to my relative	26 (86.7)	2.88 ± 1.24
*Section* *7: Motivation*		
The way that the application displays the content (texts, images, videos, audio) made me want to learn more	26 (86.7)	2.85 ± 1.16
*Section* *8: Social Learning*		
I find it useful to communicate with other users on the platform	22 (73.3)	2.32 ± 1.43
*Section* *9: Daily Tasks, the following functionalities have been helpful to me in my daily life*		
Medication calendar and reminder	16 (53.3)	2.06 ± 1.24
Appointments calendar and reminder (doctor, pharmacy, and others)	16 (53.3)	2.06 ± 1.24

Overall evaluation of pedagogical specifications	*n* (%)	

Acceptable	15 (50.0)	
Unacceptable	15 (50.0)	

SD: standard deviation.

**Table 4:** Evaluation of satisfaction specifications by sentence and overall.

Satisfaction questionnaire	*n* (%)	Mean ± SD
I have changed my attitude with my relative with dementia to a more positive one	26 (86.7)	2.92 ± 1.02
I will ask for help to any of my close relatives when taking care of my relative with dementia after using the understAID application	26 (86.7)	2.50 ± 1.27
I have more time for myself after using the understAID application	26 (86.7)	2.12 ± 0.91
I think that in general the understAID application is usefulness for me and my relative	28 (93.3)	2.79 ± 1.13
In general, I feel satisfied with the understAID application	28 (93.3)	3.25 ± 1.04

Overall evaluation of satisfaction	*n* (%)	

Acceptable	10 (33.3)	
Unacceptable	20 (66.7)	

SD: standard deviation.

**Table 5:** Impact on the caregiver after using the understAID application.

	Control group (*n* = 31)				Experimental group (*n* = 30)			
	Preintervention (mean ± SD)	Postintervention (mean ± SD)	*t*	*P* value	Preintervention (mean ± SD)	Postintervention (mean ± SD)	*t*	*P* value
CES-D	21.42 ± 8.64	20.77 ± 9.02	0.548	0.587	19.40 ± 9.03	17.03 ± 7.07	2.180	0.037^*∗*^
CCS	11.03 ± 2.24	10.97 ± 2.60	0.135	0.893	11.93 ± 2.61	11.70 ± 2.18	0.754	0.457
RCCS	19.55 ± 6.40	19.10 ± 5.71	0.644	0.525	18.67 ± 4.90	18.60 ± 4.75	0.107	0.916

CES-D: Center for Epidemiologic Studies Depression Scale, CCS: Caregiver Competence Scale, RCCS: Revised Caregiving Satisfaction Scale, and SD: standard deviation.

^*∗*^Statistically significant differences, *P* < 0.05.
